# Understanding of the prevalence of depression in a sample of gifted children by identifying the developmental trajectory of risk and protective factors

**DOI:** 10.1192/j.eurpsy.2021.257

**Published:** 2021-08-13

**Authors:** L. Vaivre-Douret, S. Hamdioui

**Affiliations:** 1 Division of Medicine Paris Descartes, Université de Paris, Faculty of Health, Paris, France; 2 University of Paris-Saclay, UVSQ, Villejuif, National Institute of health and Medical Research (INSERM UMR 1018-CESP), Paris, France; 3 (Institut Universitaire de France, IUF), University Institute of France, Paris, France; 4 Department of Child Psychiatry, Assistante Publique-Hôpitaux de Paris (AP-HP. Centre), Necker-Enfants Malades University hospital, Paris, France; 5 Department of Endocrinology, IMAGINE Institute, Necker-Enfants Malades Université de Paris, Paris, France; 6 Faculty of Society and Humanity, Division of Psychology, Université de Paris, Paris, France

**Keywords:** Depression, Gifted children, Motor skills disorder, Learning disabilities, Social relationship

## Abstract

**Introduction:**

Developmental studies in infancy remain rare. Studies measuring depressive symptoms in gifted children are contradictory, considering more anxiety or depression than in non-gifted children. Furthermore, questionnaires or anxiety scales are used without taking into account all aspects of mood disorders and thus, rarely depression scales have been conducted.

**Objectives:**

To refine the developmental trajectory of depression in a national sample of French gifted children by identification of the specific risk and protective factors.

**Methods:**

A self-reported depression scale MDI-C (Multiscore-Depression-Inventory-for-Children) were sent to families to be administered to their gifted children from preschool to high school, aged from 4 to 20 years-old (IQ >125) looking for help from gifted associations. A larger wave of data collection on different aspects of child and family history was collected (pregnancy, term and delivery mode, neonatal period, psychomotor development, health, schooling, interpersonal relationships with family and friends, personality, parental socio-economic status).

**Results:**

438 children (> 130) were eligible. Regarding anamnestic fields, Exploratory-Factor-Analysis highlighted six predictive factors of depression with eigenvalues from 1.09 to 3.17. Major factors explaining 62.96% of total variance are: Factor-1 “motor skills disorder” (14.53%). Factor-2 “positive family relationships” (14.04%). Factor-3 “positive social relationships with peers” (14.02%). Factor-4 “integration of social codes” (11.23%). Factor-6 “Learning disabilities and rehabilitation” (10.1%).

**Conclusions:**

Our findings highlight specifics risk factors of depression in the field of learning disabilities or social cognition, while a good quality of social relationships since childhood seems to be a preventive factor.

**Disclosure:**

No significant relationships.

